# The role of NLRP3 inflammasome in multiple sclerosis: pathogenesis and pharmacological application

**DOI:** 10.3389/fimmu.2025.1572140

**Published:** 2025-04-02

**Authors:** Wen-Gang Zhang, Xiao-Rui Zheng, Yi Yao, Wei-Jia Sun, Bo-Zong Shao

**Affiliations:** ^1^ The First Medical Center, General Hospital of the Chinese People’s Liberation Army, Beijing, China; ^2^ Medical Supplies Center, General Hospital of the Chinese People’s Liberation Army, Beijing, China

**Keywords:** multiple sclerosis, NLRP3 inflammasome, inflammation, experimental autoimmune encephalomyelitis, autophagy

## Abstract

Multiple sclerosis (MS) is widely acknowledged as a chronic inflammatory autoimmune disorder characterized by central nervous system (CNS) demyelination and neurodegeneration. The hyperactivation of immune and inflammatory responses is recognized as a pivotal factor contributing to the pathogenesis and progression of MS. Among various immune and inflammatory reactions, researchers have increasingly focused on the inflammasome, a complex of proteins. The initiation and activation of the inflammasome are intricately involved in the onset of MS. Notably, the NLRP3 inflammasome, the most extensively studied member of the inflammasome complex, is closely linked with MS. This review will delve into the roles of the NLRP3 inflammasome in the pathogenesis and progression of MS. Additionally, therapeutic strategies targeting the NLRP3 inflammasome for the treatment of MS, including natural compounds, autophagy regulators, and other small molecular compounds, will be detailed in this review.

## Introduction

Multiple Sclerosis (MS) is a chronic, progressive, and frequently disabling neurological disorder that affects the central nervous system (CNS), which comprises the brain and spinal cord (1–3). This condition is characterized by the destruction of myelin, the protective sheath surrounding nerve fibers, leading to disrupted communication between the brain and the rest of the body (4, 5). Demyelination in MS results in a diverse array of symptoms, including motor, sensory, and cognitive impairments. The etiology of MS is multifaceted, involving a complex interaction between genetic predisposition and environmental factors (6, 7). Histopathologically, MS lesions or plaques are characterized by focal demyelination, gliosis, and axonal loss (8, 9). These lesions can occur throughout the CNS but are commonly found in the periventricular regions, optic nerves, brainstem, and spinal cord (10, 11). The pathogenesis of MS involves an autoimmune and inflammatory response targeting CNS components (12–14). Therefore, modulating the overactivation of immune and inflammatory responses represents a promising therapeutic strategy against MS (15–19). In this paper, we will focus on the role of the NLRP3 inflammasome, a crucial component of the innate immune system, in the pathogenesis and progression of MS. Additionally, we will discuss the pharmacological application of agents that inhibit the NLRP3 inflammasome.

To gather relevant studies, we conducted a comprehensive literature search on PubMed (https://pubmed.ncbi.nlm.nih.gov/) and Web of Science (https://www.webofscience.com/), concentrating on publications from the past two decades. Our search terms were “NLRP3 inflammasome, multiple sclerosis, experimental autoimmune encephalomyelitis, natural compound, autophagy, small molecular compound, inflammation, mechanism, therapy”. We prioritized studies published in the last ten years. The process involved initially screening titles and abstracts, then obtaining full-text articles for in-depth analysis.

## Part I: NLRP3 inflammasome in MS: pathogenesis and progression

### Assembly and activation of the NLRP3 inflammasome

The inflammasome, a critical inducer of the innate immune response, plays a pivotal role in recognizing and targeting numerous invasive or internal pathogens (20–22). It is widely recognized that inflammasomes are primarily produced in immune and inflammatory cells, including macrophages, T lymphocytes, and natural killer (NK) cells, thereby contributing to the initiation of anti-pathogen immune inflammatory responses (23–25). To date, several types of inflammasomes have been identified, notably including NLRP1, NLRP2, NLRP3, double-stranded DNA sensors absent in melanoma 2 (AIM2), and NLRC4 inflammasomes (26–28). The NLRP3 inflammasome, compared to other inflammasomes, is the most well-studied and has been linked to various diseases, including MS (29–31). It significantly contributes to MS development through pro-inflammatory cytokine production and interactions with other immune responses. This section details the NLRP3 inflammasome’s components, activation mechanisms, and its importance in MS.

The NLRP3 inflammasome consists of three components: the NLRP3 protein, the adapter protein apoptosis-associated speck-like protein (ASC), and procaspase-1 (32, 33). In the absence of activating factors such as pathogen-associated molecular patterns (PAMPs) and danger-associated molecular patterns (DAMPs), the leucine-rich repeats (LRRs) and NACHT domain in the NLRP3 protein interact tightly, preventing the interaction between the NLRP3 protein and ASC (34–36). Upon exposure to immune stimuli, the NLRP3 protein is activated, facilitating its interaction with ASC and procaspase-1 via the pyrin domain (PYD) and caspase recruitment domain (CARD), respectively, leading to the assembly of the NLRP3 inflammasome (37–39).

As previously described by our group and other researchers, the activation of the NLRP3 inflammasome involves two distinct steps (29, 40–42). In the initial step, priming signals are triggered by specific PAMPs or DAMPs acting on Toll-like receptor 4 (TLR4), resulting in the activation of the NF-κB-mediated pathway. This activation enhances the transcription of NLRP3 inflammasome-related components, including the NLRP3 protein, pro-interleukin-1β (proIL-1β), and proIL-18. In the subsequent second step, further stimulation of immune and inflammatory cells leads to the oligomerization of the NLRP3 protein, followed by the assembly of the NLRP3 protein, ASC, and procaspase-1 into the NLRP3 inflammasome complex. The successful formation of the NLRP3 inflammasome catalyzes the conversion of procaspase-1 to caspase-1, which subsequently processes proIL-1β and proIL-18 into their mature forms, IL-1β and IL-18. These mature cytokines are secreted, initiating a cascade of immune or inflammatory reactions.

Several factors have been identified as activators of the NLRP3 inflammasome. Lipopolysaccharide (LPS) is widely recognized as a classic ligand for the activation of TLR4, initiating the first step of NLRP3 inflammasome activation (43, 44). Additionally, various factors have been shown to effectively induce the second step of NLRP3 inflammasome activation, including adenosine triphosphate (ATP, which triggers intracellular K^+^ efflux), PAMPs, DAMPs, silica, β-amyloid, autophagy deficiency, and factors leading to mitochondrial Ca^2+^ overload (45–49).

### Relationship between the NLRP3 inflammasome and MS

The NLRP3 inflammasome has been implicated in the pathogenesis and progression of various diseases, including cardiovascular conditions (such as atherosclerosis and myocardial infarction) (50–52), digestive disorders (such as inflammatory bowel disease and pancreatic disease) (53–55), malignancies (56–58), and metabolic diseases (such as diabetes and obesity) (59–61). In the context of multiple sclerosis (MS), the role of the NLRP3 inflammasome has been extensively studied. Elevated IL-1β levels have been identified in the serum of patients with primary progressive MS through RNA sequencing. Notably, primary progressive MS patients with high IL-1β gene expression levels in peripheral blood mononuclear cells exhibited significantly faster disease progression compared to those with low IL-1β levels (62, 63). Further studies demonstrated that the NLRP3 inflammasome was overactivated in monocytes from patients with primary progressive MS (62, 63). These findings suggested that IL-1β and the NLRP3 inflammasome could serve as prognostic biomarkers and potential therapeutic targets in primary progressive MS.

Variants in the NLRP3 inflammasome have also been associated with the susceptibility and severity of MS. Single nucleotide polymorphisms and expression levels of NLRP3 are closely related to susceptibility to relapsing-remitting MS (64–66). A pilot study reported overexpression of NLRP3 inflammasome components, including NF-κB, NLRP3, and caspase-1, in the serum during the early stages of MS (67). Moreover, bioinformatics analyses have suggested that the NLRP3 inflammasome-related NLR signaling pathway may play a critical role in COVID-19-related MS (68). These findings underscore a close relationship between the NLRP3 inflammasome and MS.

Despite the growing body of research on this topic, the mechanisms and influencing factors of the NLRP3 inflammasome in the pathogenesis and progression of MS are not fully understood. A recent study using an experimental autoimmune encephalomyelitis (EAE) mouse model, a widely accepted model for MS, demonstrated that NLRP3 exacerbated EAE severity through ROS-dependent neutrophil extracellular trap (NET) formation in the brain (69). NLRP3 facilitated NET formation in a ROS-dependent and PAD4-independent manner in brain-infiltrated neutrophils. Additionally, the NLRP3 inflammasome in microglial cells has been shown to contribute to demyelination and neurodegeneration induced by the neuronal accumulation of peroxidated lipids (70). The NLRP3 inflammasome has also been implicated in TRPM2 and IL-11-mediated neuroinflammation and cognitive deficits in a cuprizone-induced MS model (71, 72).

## Part II: pharmacological application of MS treatment targeting the NLRP3 inflammasome

**Figure 1:**
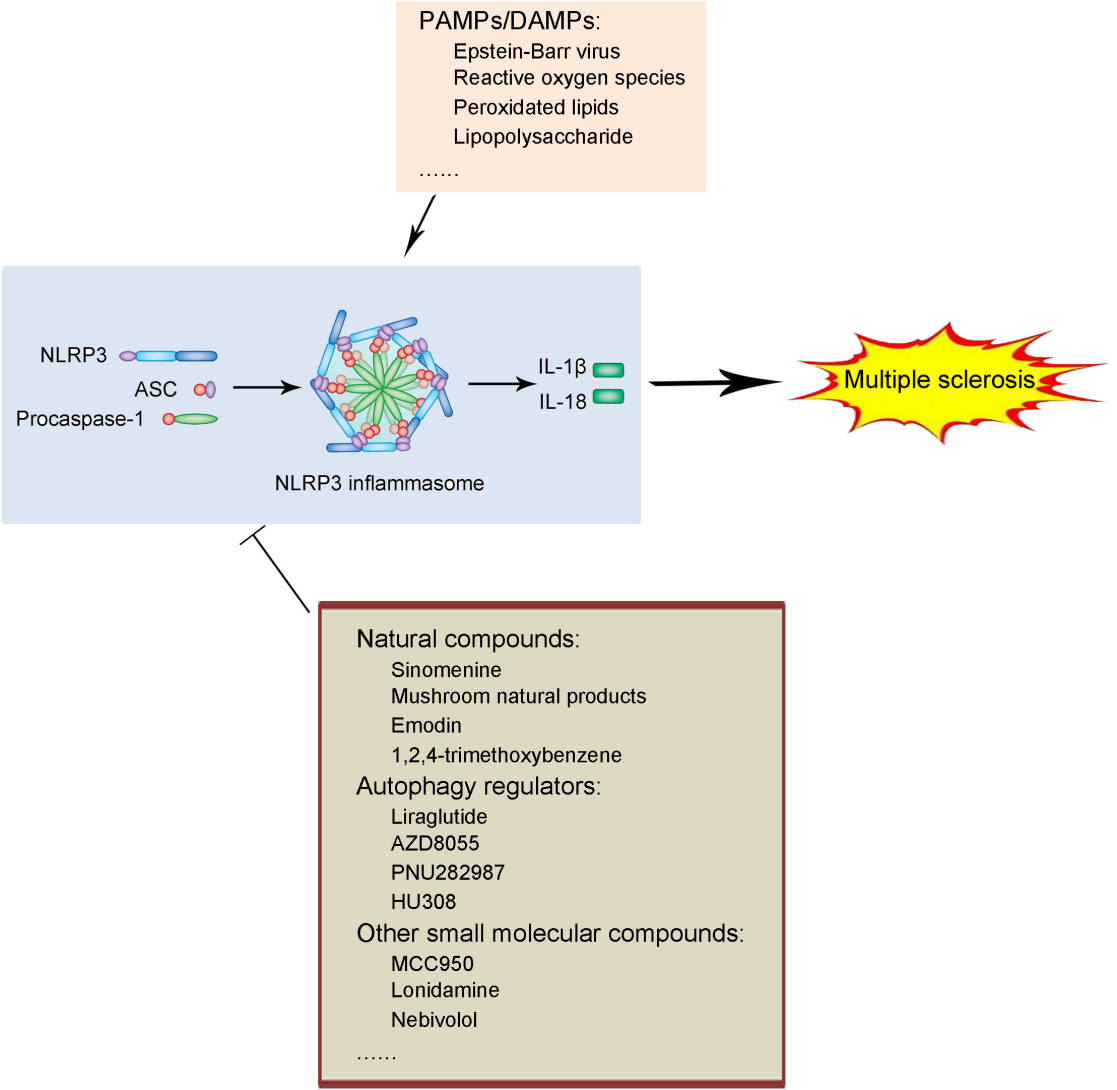
Schematic illustration of biological process of NLRP3 inflammasome and pharmacological application of NLRP3 inflammasome inhibition in multiple sclerosis treatment. Under the exposure of PAMPs/DAMPs including Epstein-Barr virus, reactive oxygen species, peroxidated lipids, and lipopolysaccharide, the NLRP3 protein as well as proIL-1β and proIL-18 production. The assembly and formation of the NLRP3 inflammasome through the combination of the NLRP3 protein, ASC and procaspase-1 were assembly, which triggered the formation and activation of the NLRP3 inflammasome. The NLRP3 inflammasome catalyzes the maturation and secretion of IL-1β and IL-18. The production and release of IL-1β and IL-18 contributed to the pathogenesis and progression of multiple sclerosis. The process of NLRP3 inflammasome activation can be blocked by several kinds of NLRP3 inflammasome inhibitors in multiple sclerosis, including natural compounds (sinomenine, mushroom natural products, emodi, and 1,2,4-trimethoxybenzene), autophagy regulators (liraglutide, AZD8055, PNU282987, and HU308), and other small molecular compounds (MCC950, lonidamine, and nebivolol), which serve as potential therapeutic strategies against multiple sclerosis.

As discussed above, the NLRP3 inflammasome is closely related to the pathogenesis and progression of multiple sclerosis (MS), suggesting that it is a promising target for therapeutic intervention. Numerous studies have reported various agents that appear effective in attenuating MS by inhibiting the NLRP3 inflammasome. Several popular and well-studied agents will be introduced and discussed in detail in the following sections (illustrated in **Figure 1** and listed in **Table 1**).

**Table 1:** Potential pharmacological application of NLRP3 inflammasome inhibition in multiple sclerosis treatment.

NLRP3 inflammasome inhibitors		Potential pharmacological mechanisms	Reference
Type	Name		
Natural compounds	Sinomenine	Alleviating microglial and astrocytic mobilization, demyelination, and axonal damage	(76)
	Mushroom natural products	Suppressing the NLRP3 inflammasome activation and oligomerization	(77)
	Emodin	Regulating the SIRT1/PGC-1α/NLRP3 signaling pathway	(78)
	1,2,4-trimethoxybenzene	Inhibiting ASC and protein-protein interaction between NLRP3 and ASC	(79)
Autophagy regulators	Liraglutide	Regulating the pAMPK and AMPK/SIRT1 signaling pathways	(88, 89)
	AZD8055	Regulating the mammalian target of mTOR/ROS/NLRP3 pathway	(90)
	PNU282987	Activating α7nAChR	(91)
	HU308	Activating CB2R	(92)
Other small molecular compounds	MCC950	Suppressing the activation of glial cells; preventing microglia polarization to M1 phenotype	(93–96)
	Lonidamine	Binding to the NLRP3 inflammasome ligand ASC and inhibiting its oligomerization	(97)
	Nebivolol	Regulating the M1/M2 polarization	(98)

### Natural compounds

Natural compounds, also known as natural extracts, are substances isolated or derived from organisms such as plants, animals, microorganisms, and humans (73–75). Some of these compounds have demonstrated potential in alleviating MS symptoms by targeting the NLRP3 inflammasome. For example, Kiasalari et al. (76) revealed that sinomenine, a natural alkaloid with various therapeutic benefits including anti-inflammatory and immunosuppressive activities, decreased EAE severity. This effect was attributed to its reduction of microglial and astrocytic activation, demyelination, and axonal damage, along with its suppression of neuroinflammation. Additionally, various mushroom-derived natural products have been shown to exert neuroprotective effects in MS through novel high-throughput screening methods by suppressing NLRP3 inflammasome activation and oligomerization (77). Cui et al. (78) demonstrated that emodin, a compound extracted from herbs such as rhubarb, could improve symptoms of experimental autoimmune encephalomyelitis, potentially by regulating the silent information regulator of transcription 1 (SIRT1)/peroxisome proliferator-activated receptor gamma coactivator 1-alpha (PGC-1α)/NLRP3 signaling pathway and inhibiting microglial inflammation. Furthermore, 1,2,4-trimethoxybenzene, an active ingredient in essential oils, significantly ameliorated EAE progression and demyelination by inhibiting ASC and the protein-protein interaction between NLRP3 and ASC (79). However, despite the promising potential of these natural compounds in preclinical studies, few have been successfully applied in clinical practice for the treatment of MS. Therefore, further research is needed to explore and validate the therapeutic efficacy of natural compounds targeting the NLRP3 inflammasome in MS.

### Autophagy regulators

Autophagy is a fundamental catabolic cellular process responsible for degrading protein aggregates and damaged organelles into metabolic components through lysosomal recycling, thereby maintaining cellular homeostasis and vitality (75, 80–82). This process is ubiquitously present in virtually all cell types and is evolutionarily conserved from yeast to mammals (83, 84). In recent years, extensive research has explored the role of autophagy in inflammation- and immune-related diseases through its regulatory effects on inflammatory and immune responses (85–87). Several autophagy regulators have shown potential in alleviating MS by modulating the NLRP3 inflammasome. Liraglutide, a glucagon-like peptide-1 receptor (GLP-1R) agonist, has been shown to ameliorate central nervous system demyelination and inflammation in EAE models by regulating the pAMPK and AMPK/SIRT1 signaling pathways (88, 89). Additionally, He et al. (90) reported that AZD8055, an autophagy activator, could reduce EAE severity through anti-inflammatory and anti-pyroptotic effects via the mammalian target of rapamycin (mTOR)/ROS/NLRP3 pathway. Our previous studies have further elucidated the role of autophagy in MS. We demonstrated that activating the α7 nicotinic acetylcholine receptor (α7nAChR) with PNU282987 could alleviate neuroinflammation in EAE models by enhancing monocyte/microglia autophagy, thereby inhibiting the NLRP3 inflammasome (91). Another study conducted by our group revealed that activation of cannabinoid receptor 2 (CB2R) with HU308 protected against neuroinflammation through autophagy-mediated suppression of the NLRP3 inflammasome. This effect was mediated via the autophagy-related gene 5 (ATG5)-dependent signaling pathway (92).

### Other small molecular compounds

In addition to natural compounds and autophagy regulators, several other small molecular compounds have been demonstrated to effectively alleviate MS by inhibiting the NLRP3 inflammasome. For instance, MCC950, a potent and selective small-molecule inhibitor of NLRP3, was first reported in 2015 to alleviate MS, as well as type 2 diabetes, Alzheimer’s disease, atherosclerosis, and cryopyrin-associated periodic syndrome (CAPS), by blocking both canonical and noncanonical NLRP3 activation at nanomolar concentrations (93). Subsequent studies have shown that MCC950 can reduce neurological impairment in EAE models by inhibiting the NLRP3 inflammasome, thereby suppressing glial cell activation and preventing the polarization of microglia to the pro-inflammatory M1 phenotype (94–96). Additionally, lonidamine (LND), a small-molecule inhibitor of glycolysis used as an antineoplastic drug, has been evidenced to have anti-inflammatory effects. LND was reported to alleviate EAE by directly binding to the NLRP3 inflammasome component ASC and inhibiting its oligomerization (97). Another small molecular compound shown to alleviate MS through NLRP3 inflammasome inhibition is nebivolol, which has been found to modulate M1/M2 microglial polarization (98). These findings highlight the therapeutic potential of small molecular compounds in targeting the NLRP3 inflammasome for the treatment of MS. Further research is warranted to explore the clinical applications and efficacy of these agents in MS patients.

## Conclusion

In conclusion, recent studies have highlighted the critical roles of the NLRP3 inflammasome in the pathogenesis and progression of MS (**Figure 1**). We are fortunate to have a variety of inhibitors targeting NLRP3 inflammasome activation that have demonstrated efficacy in alleviating MS symptoms. These include natural compounds, autophagy regulators, and other small molecular compounds. Despite progress, significant hurdles remain in applying NLRP3 inflammasome-targeted therapies clinically, especially concerning long-term safety and side effects. Few agents have been successfully used in MS treatment due to insufficient evidence for long-term safety and potential risks of side effects associated with prolonged use of such agents. Therefore, further research is essential to develop effective MS therapies by targeting the NLRP3 inflammasome.
